# Crystal structure, Hirshfeld surface analysis and DFT calculations of 7-bromo-2,3-di­hydro­pyrrolo[2,1-*b*]quinazolin-9(1*H*)-one

**DOI:** 10.1107/S2056989022007800

**Published:** 2022-08-09

**Authors:** Akmaljon Tojiboev, Burkhon Elmuradov, Nuritdin Kattaev, Asqar Abdurazakov, Azizbek Nasrullayev, Bakhodir Tashkhodjaev

**Affiliations:** a University of Geological Sciences, Olimlar street, 64, Mirzo Ulugbek district, Tashkent, Uzbekistan; b Department of Chemistry, National University of Uzbekistan named after Mirzo Ulugbek, Tashkent, Uzbekistan; c S. Yunusov Institute of Chemistry of Plant Substances, Academy of Sciences of Uzbekistan, Tashkent, Uzbekistan; d Department of Organic Synthesis and Bioorganic Chemistry, Samarkand State University, Samarkand, Uzbekistan; Vienna University of Technology, Austria

**Keywords:** crystal structure, 7-bromo-2,3-di­hydro­pyrrolo­[2,1-*b*]quinazolin-9(1*H*)-one, Hirshfeld surface analysis, DFT

## Abstract

The mol­ecular structure of the title compound, C_11_H_9_BrN_2_O, is almost planar. The benzene and pyrimidine rings are essentially coplanar, with r.m.s. deviations of 0.0130 Å, and the largest displacement is for the flap atom of the di­hydro­pyrrole moiety [0.154 (7) Å]. Hirshfeld surface analyses revealed that the crystal packing is dominated by H⋯H, Br⋯H/H⋯Br and O⋯H/H⋯O inter­actions, and Br⋯Br inter­actions in the crystal structure are also observed. Theoretical calculations using density functional theory (DFT) with the B3LYP functional basis set gave numerical parameters for the frontier molecular orbitals.

## Abstract 2

The crystal structure of the title compound has been characterized by single-crystal X-ray diffraction and Hirshfeld surface analyses. The mol­ecular structure and frontier orbitals were also investigated using DFT.

## 1. Chemical context

Quinazolines are of significant inter­est for their various biological properties (Rajput *et al.*, 2012 ▸; Ramesh *et al.*, 2012 ▸; Khan *et al.*, 2014 ▸; Ajani *et al.*, 2016 ▸). This class of compounds is considered as an attractive target for medicinal chemists, because quinazoline and its derivatives are the scaffold of several potent anti­tumor drugs, for example the well-known *erlotinib* and *gefitinib* (Sordella *et al.*, 2004 ▸; Raymond *et al.*, 2000 ▸). Besides these two drugs, the Food and Drug Administration (FDA) has approved some other quinazolines as effective anti­cancer drugs, *viz. lapatinib* and *vandetanib*. In general, the reported biological activities of quinazolines include anti­bacterial, anti-inflammatory, CNS depressant, anti­convulsant, anti­fungal, anti­malarial, anti­cancer properties, which make them inter­esting for the pharmaceutical industry (Ajani *et al.*, 2015 ▸). 
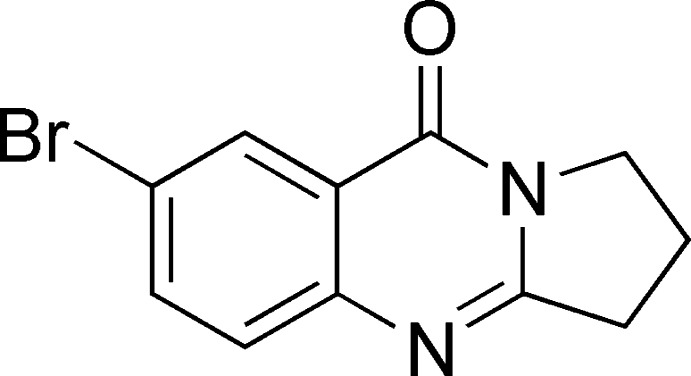


In this context, synthetic analogues of the tricyclic quin­azoline-9-one-7-bromo-2,3-di­hydro­pyrrolo­[2,1-*b*]quinazolin-9(1*H*)-one have been synthesized, amongst them the title compound with a bromine atom in position 7. In comparison with a reported literature procedure (Shakhidoyatov, 1983 ▸), this compound is now obtained in higher yields (80–88%). For this purpose, condensation of 2-amino-5-brombenzoic acid with appropriate pyrrolidin-2-one was used whereas in the literature (Shakhidoyatov, 1983 ▸), 2-amino-5-brombenzoic acid was added to the corresponding lactam mixture with a condensing agent (POCl_3_) at room temperature (293–298 K) and the reaction products separated by extraction after the reaction mixture was reduced to pH = 9–10 with NH_4_OH. As distinguished from the reported procedure, we carried out these reactions by cooling in an ice bath at a much lower temperature (273–275 K) and for a relatively longer period of time. The reaction products were finally separated by cold NH_4_OH at pH = 10–11. In general, the inter­actions of 7-bromo-2,3-di­hydro­pyrrolo­[2,1-*b*]quinazolin-9(1*H*)-one with aldehydes are well-studied (Abduraza­kov *et al.*, 2007 ▸).

Here, we report the mol­ecular and crystal structures as well as Hirshfeld surface analysis and the frontier mol­ecular orbitals calculated by density functional theory (DFT) with the B3LYP functional basis set.

## 2. Structural commentary

**Figure 1:**
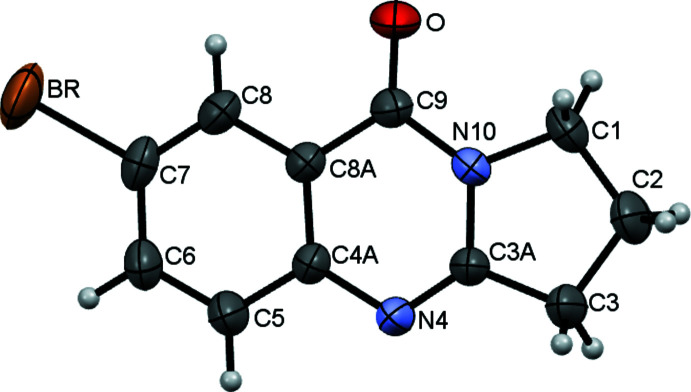
The mol­ecular structure of the title compound with displacement ellipsoids drawn at the 50% probability level.

The mol­ecular structure of the title compound is shown in Fig. 1 ▸. The mol­ecule is almost planar. In particular, the benzene and pyrimidine rings are essentially coplanar, with an r.m.s. deviations of 0.0130 Å from planarity. The remaining atoms of the di­hydro­pyrrole ring are slightly displaced from these planes, with deviations of −0.060 (5) Å for C1, −0.154 (7) Å for flap atom C2, and 0.060 (6) Å for C3. The acyclic C7—Br1 bond length 1.900 (3) Å is consistent with the data for other Br-substituted tricyclic quinazolinone derivatives (Mukarramov *et al.*, 2009 ▸; Tozhiboev *et al.*, 2007*a* ▸; D’yakonov *et al.*, 1992 ▸; Okmanov *et al.*, 2009 ▸; Pereira *et al.*, 2005 ▸).

## 3. Supra­molecular features

**Figure 2:**
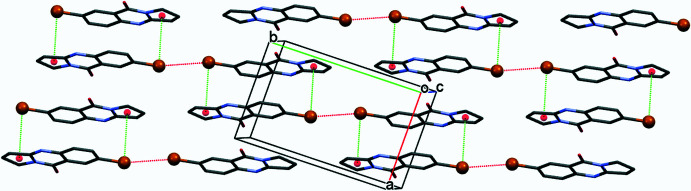
The packing of the title compound in a view perpendicular to (002). Inter­molecular Br⋯Br contacts and C—Br⋯*Cg*1 are shown as red and green dashed lines, respectively. *Cg*1 is the centroid of the C1–C3/C3*A*/N10 ring.

**Figure 3:**
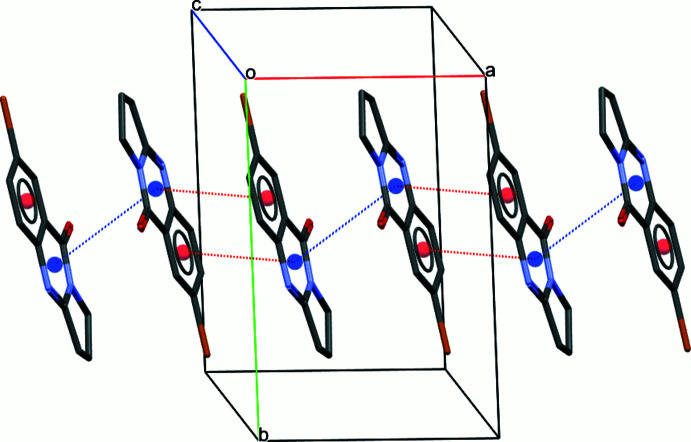
The packing of the title compound in a view approximately along [001], showing stacking between adjacent mol­ecules in terms of *Cg*2⋯*Cg*2 (blue dashed lines) and *Cg*2⋯*Cg*3 (red dashed lines) inter­actions. *Cg*2 is the centroid (blue sphere) of the pyrimidine ring and *Cg*3 is the centroid (red sphere) of the benzene ring. H atoms are omitted for clarity.

**Figure 4:**
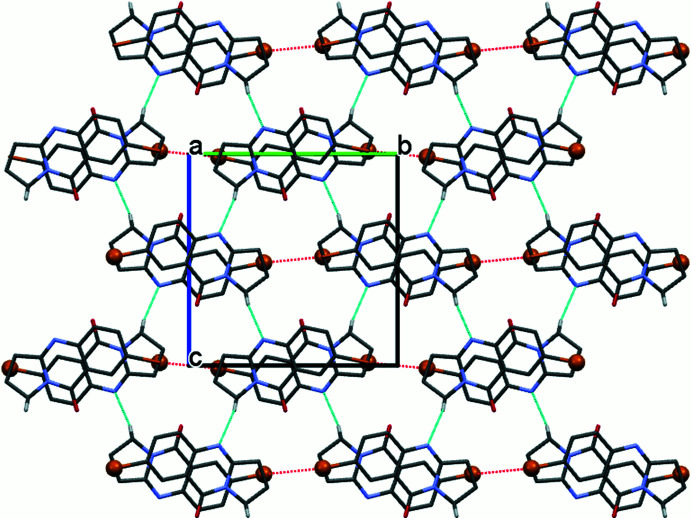
Packing of the title compound along [100], with inter­molecular C—H⋯N contacts shown as light-blue dashed lines.

In the crystal, mol­ecules participate in centrosymmetric halogen-bonding dimers with Br⋯Br inter­molecular contacts of 3.5961 (5) Å, which is shorter than the sum of van der Waals radii (Bondi *et al.*, 1964 ▸) of two bromine atoms (3.66 Å). The C7—Br⋯Br angle amounts to 166.70 (14)°. The mol­ecules also engage in weak C7—Br⋯*Cg* inter­actions, with Br⋯*Cg*1(2 − *x*, 1 − *y*, 1 − *z*) = 3.6428 (15) Å, forming a layered network (Fig. 2 ▸). Additional π–π stacking (Fig. 3 ▸) occurs between the aromatic rings of neighbouring mol­ecules, with the distance between the centroids *Cg*2⋯*Cg*2^i^ being 3.9969 (14) Å [symmetry code: (i) 1 − *x*, 1 − *y*, 1 − *z*] and a ring slippage of 1.569 Å, and *Cg*2⋯*Cg*3^ii^ being 3.7513 (16) Å [symmetry code: (ii) 2 − *x*, 1 − *y*, 1 − *z*] and a ring slippage of 1.194 Å. Both short inter­molecular contacts help to stack parallel mol­ecules along [100]. The resulting two-dimensional network extends parallel to (002), with neighbouring layers linked through C1—H1*B*⋯N4 short inter­molecular contacts, H1*B*⋯N4(*x*, 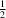 − *y*, 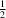 + *z*) = 2.73 Å, C1—H1*B*⋯N4(*x*, 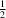 − *y*, 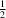 + *z*) = 169°, to form the full three-dimensional structure (Fig. 4 ▸).

## 4. Hirshfeld surface analysis

**Figure 5:**
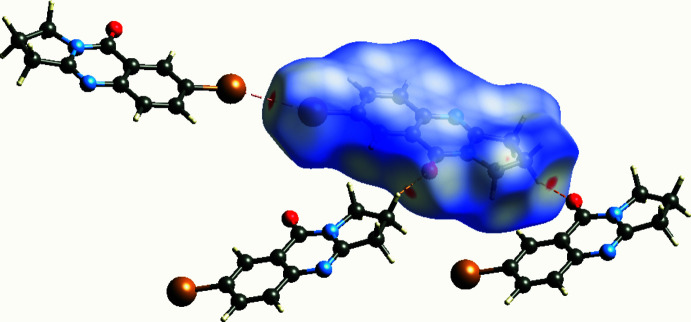
The Hirshfeld surface of the title compound mapped over *d*_norm_, showing the close contacts.

**Figure 6:**
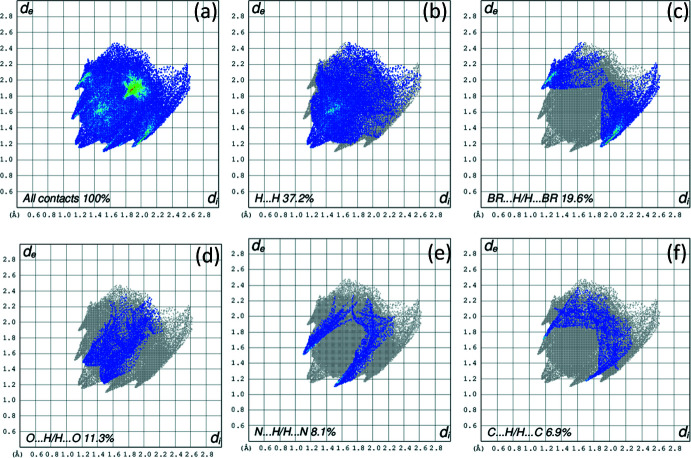
A view of the two-dimensional fingerprint plots for the title compound, showing (*a*) all inter­actions, and delineated into (*b*) H⋯H, (*c*) Br⋯H/H⋯Br, (*d*) O⋯H/H⋯O, (*e*) N⋯H/H⋯N and (*f*) C⋯H/H⋯C inter­actions. The *d*_i_ and *d*_e_ values are the closest inter­nal and external distances (in A°) from given points on the Hirshfeld surface contacts.

In order to qu­antify the inter­molecular inter­actions in the crystal of the title compound, a Hirshfeld surface (HS) analysis (Spackman *et al.*, 2009 ▸) was performed and associated two-dimensional fingerprint plots (McKinnon *et al.*, 2007 ▸) were generated with the program *CrystalExplorer* (Spackman *et al.*, 2021 ▸). The HS mapped over *d*_norm_ is depicted in Fig. 5 ▸, which shows the most prominent inter­molecular inter­actions as red spots corresponding to the Br⋯Br, C—H⋯O and N—H⋯O contacts. The two-dimensional fingerprint plot for all contacts is given in Fig. 6 ▸ *a.* H⋯H contacts are responsible for the largest contribution (37.2%) to the Hirshfeld surface (Fig. 6 ▸ *b*). Besides these contacts, Br⋯H/H⋯Br (19.6%), O⋯H/H⋯O (11.3%), N⋯H/H⋯N (8.1%) and C⋯H/H⋯C (6.9%) inter­actions contribute significantly to the total Hirshfeld surface; their decomposed fingerprint plots are shown in Fig. 6 ▸ *c*–*f*. The contributions of further contacts are only minor and amount to N⋯C/C⋯N (3.5%), O⋯C/C⋯O (2.0%), Br⋯C/C⋯Br (0.9%), Br⋯Br (0.8%), O⋯N/N⋯O (0.5%) and Br⋯N/N⋯Br (0.3%).

## 5. Frontier mol­ecular orbitals

**Figure 7:**
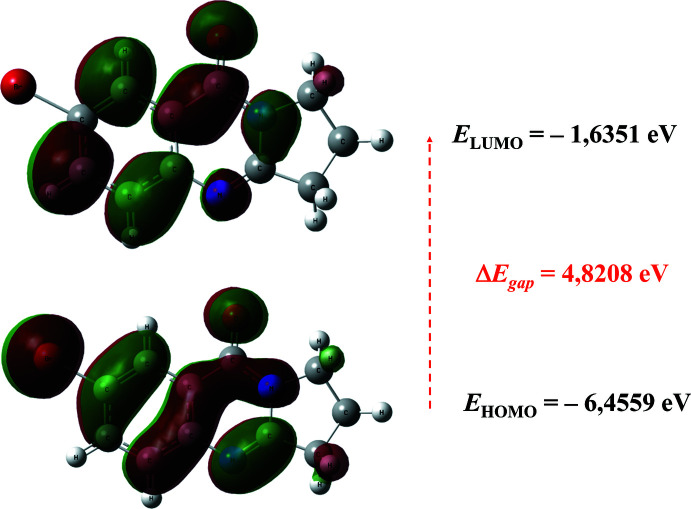
The frontier mol­ecular orbitals (HOMO-LUMO) and the resulting band gap of the title mol­ecule.

DFT was used to calculate the frontier mol­ecular orbitals (FMOs, Fig. 7 ▸), which give important details of how a mol­ecule inter­acts with other species, for example in terms of mol­ecular reactivity and the ability of a mol­ecule to absorb light. From the highest occupied mol­ecular orbital (HOMO) electrons can be donated to the lowest unoccupied mol­ecular orbital (LUMO). Moreover, the energy of the HOMO is directly related to the ionization potential, while the LUMO energy is directly related to the electron affinity, and the resulting energy difference (or energy gap) between HOMO and LUMO gives information about the stability of a mol­ecule. In the case where the energy gap is small, the mol­ecule is highly polarizable and has a high chemical reactivity. By using the HOMO and LUMO energy values of a mol­ecule, its electronegativity (*c*), chemical hardness (*h*) and chemical softness (*s*) can be calculated as follows: *c* = (*I* + *A*)/2; *h* = (*I - A*)/2; *s* = 1/2*h*, where *I* and *A* are the ionization potential and electron affinity, respectively, where *I* = –*E*_HOMO_ and *A* = –*E*_LUMO_ (Pir *et al.*, 2014 ▸; Azizov *et al.*, 2021 ▸).

*E*_HOMO_ and *E*_LUMO_, electronegativity (*c*), hardness (*h*), potential (*m*), electrophilicity (*w*) and softness (*s*) for the title mol­ecule were calculated at the DFT/B3LYP level using the 6-311++G(d,p) basis set (Table 1 ▸). The values of *h* and *s* are significant for the evaluation of both reactivity and stability. The electron transition from the HOMO to the LUMO energy level is shown in Fig. 7 ▸. The energy band gap [Δ*E* = *E*_LUMO_ − *E*_HOMO_] of the mol­ecule is 4.8208 eV, the frontier mol­ecular orbital energies *E*_HOMO_ and *E*_LUMO_ being −6.4559 and −1.6351 eV, respectively. The high value of the band gap (4,8208 eV) indicates the relatively high stability of the title mol­ecule.

## 6. Database survey

A search in the Cambridge Structural Database (CSD, version 2022; Groom *et al.*, 2016 ▸) gave four matches of mol­ecules containing the 2,3-di­hydro­pyrrolo­[2,1-*b*]quinazolin-9(1*H*)-one moiety with a similar conformation to that in the title structure: de­oxy­vasicinone (TEFGEQ; Turgunov *et al.*, 1995 ▸), de­oxy­vasicinonium chloride (TEFGIU; Turgunov *et al.*, 1995 ▸), bis­(de­oxy­vasicinonium) tetra­chlorido­cobaltate(II) (TEFGOA; Turgunov *et al.*, 1995 ▸) and 4-oxo-2,3-tetra­methyl­ene-3,4-di­hydro­quinazolinium 2,3-tetra­methyl­ene-3,4-di­hydro­quinazol-4-one hemikis(oxalate) oxalic acid solvate (TITGUZ; Tozhiboev *et al.*, 2007*b* ▸). A search for compounds substituted in position 7 of 2,3-di­hydro­pyrrolo­[2,1-*b*]quinazolin-9(1*H*)-one moiety gave only two hits: *N*-(9-oxo-1,2,3,9-tetra­hydro­pyrrolo­[2,1-*b*]quinazolin-7-yl)propanamide sesquihydrate (GABJAX; Elmuradov *et al.*, 2016 ▸) and 3*b*-hy­droxy-7-meth­oxy-2,3-di­hydro­pyrrolo­[2,1-*b*]quinazolin-9(1*H*)-one mono­hydrate (HIHLIT; Magotra *et al.*, 1996 ▸). Comparing the listed structures with that of the title compound gave analogous complanarities of the benzene and pyrimidine rings. In the case of structures TEFGEQ, GABJAX and HIHLIT they have also similarities regarding π–π stacking inter­actions.

## 7. Synthesis and crystallization

**Figure 8:**
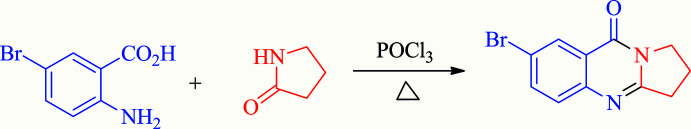
The reaction scheme of the title compound.

The reaction scheme to yield the title compound is shown in Fig. 8 ▸. To a mixture of 4.32 g (20 mmol) 2-amino-5-bromo­benzoic acid and 2.72 g (32 mmol) pyrrolidin-2-one, 21.8 g (13 ml) (*d* = 1.675) (0.142 mol) of phospho­roxychloride were added dropwise over 1 h at 273–275 K. The reaction mixture was then heated at 368–371 K for 2 h, it was subsequently cooled and finally poured over ice. The temperature of the mixture was kept at around 273–275 K. When the reaction mixture was completely decomposed, it was brought to pH = 10–11 with 25%_wt_ ammonium hydroxide solution. The light-yellow precipitate was filtered off, dried and recrystallized from methanol. The yield of the product was 4.35 g (82%), m.p. 431–433 K (literature, m.p. = 430–431 K; Shakhidoyatov, 1983 ▸).

**^1^H NMR (400 Mz, CDCl_3_, δ, ppm)**: 8.4 (1H, *d*, *J* = 2.4, H-8), 7.8 (1H, *dd*, *J* = 2.4, *J* = 8.8, H-6), 7.5 (1H, *d*, *J* = 8.8, H-5), 4.2 (2H, *q*, *J* = 7.2, H-1), 3.18 (2H, *t*, *J* = 7.6, H-3), 2.31 (2H, *m*, H-2).

## 8. Refinement

Crystal data, data collection and structure refinement details are summarized in Table 2 ▸. H atoms attached to C were positioned geometrically, with C—H = 0.93 Å (for aromatic) or C—H = 0.97 Å (for methyl­ene H atoms), and were refined with *U*_iso_(H) = 1.2*U*_eq_(C).

## Supplementary Material

Crystal structure: contains datablock(s) I. DOI: 10.1107/S2056989022007800/wm5655sup1.cif

Structure factors: contains datablock(s) I. DOI: 10.1107/S2056989022007800/wm5655Isup3.hkl

Supporting information file. DOI: 10.1107/S2056989022007800/wm5655Isup3.cml

CCDC reference: 2194365

Additional supporting information:  crystallographic information; 3D view; checkCIF report

## Figures and Tables

**Table 1:** Calculated parameters of the title mol­ecule calculated at the B3LYP/6–311++G(d,p) level

Parameters	DFT/B3LYP
Total energy *TE* (a.u.)	−3183.662028
*E*_HOMO_ (eV)	−6.4559
*E*_LUMO_ (eV)	−1.6351
Energy gap, Δ*E* (eV)	4.8208
Dipole moment, μ (Debye)	4.6478
Ionization potential, *I* (eV)	6.4559
Electron affinity, *A*	1.6351
Electronegativity, χ	4.0455
Hardness, η	2.4104
Electrophilicity index, ω	3.3949
Softness, σ	0.2074

**Table 2:** Experimental details

Crystal data	
Chemical formula	C_11_H_9_BrN_2_O
*M*_r_	265.11
Crystal system, space group	Monoclinic, *P*2_1_/*c*
Temperature (K)	296
*a*, *b*, *c* (Å)	7.5654 (3), 11.4972 (2), 12.1025 (3)
β (°)	105.583 (3)
*V* (Å^3^)	1013.99 (5)
*Z*	4
Radiation type	Cu *K*α
μ (mm^−1^)	5.30
Crystal size (mm)	0.45 × 0.10 × 0.10

Data collection	
Diffractometer	XtaLAB Synergy, Single source at home/near, HyPix3000
Absorption correction	Multi-scan (*CrysAlis PRO*; Rigaku OD, 2020 ▸)
*T*_min_, *T*_max_	0.400, 1.000
No. of measured, independent and observed [*I* > 2σ(*I*)] reflections	9036, 1959, 1770
*R*_int_	0.035
(sin θ/λ)_max_ (Å^−1^)	0.615

Refinement	
*R*[*F* ^2^ > 2σ(*F* ^2^)], *wR*(*F* ^2^), *S*	0.036, 0.099, 1.08
No. of reflections	1959
No. of parameters	137
H-atom treatment	H-atom parameters constrained
Δρ_max_, Δρ_min_ (e Å^−3^)	0.61, −0.56

Computer programs: *CrysAlis PRO* (Rigaku OD, 2020 ▸), *SHELXS* (Sheldrick, 2008 ▸), *SHELXL* (Sheldrick, 2015 ▸), *PLATON* (Spek, 2020 ▸) and *publCIF* (Westrip, 2010 ▸).

## supplementary crystallographic information

### Crystal data

**Table:**

C_11_H_9_BrN_2_O	*F*(000) = 528
*M**_r_* = 265.11	*D*_x_ = 1.737 Mg m^−^^3^
Monoclinic, *P*2_1_/*c*	Cu *K*α radiation, λ = 1.54184 Å
*a* = 7.5654 (3) Å	Cell parameters from 5905 reflections
*b* = 11.4972 (2) Å	θ = 3.8–71.3°
*c* = 12.1025 (3) Å	µ = 5.30 mm^−^^1^
β = 105.583 (3)°	*T* = 296 K
*V* = 1013.99 (5) Å^3^	Prismatic, colourless
*Z* = 4	0.45 × 0.10 × 0.10 mm

### Data collection

**Table:**

XtaLAB Synergy, Single source at home/near, HyPix3000 diffractometer	1959 independent reflections
Radiation source: micro-focus sealed X-ray tube	1770 reflections with *I* > 2σ(*I*)
Detector resolution: 10.0000 pixels mm^-1^	*R*_int_ = 0.035
ω scans	θ_max_ = 71.5°, θ_min_ = 5.4°
Absorption correction: multi-scan (*CrysAlisPro*; Rigaku OD, 2020)	*h* = −9→9
*T*_min_ = 0.400, *T*_max_ = 1.000	*k* = −14→13
9036 measured reflections	*l* = −14→14

### Refinement

**Table:**

Refinement on *F*^2^	Hydrogen site location: inferred from neighbouring sites
Least-squares matrix: full	H-atom parameters constrained
*R*[*F*^2^ > 2σ(*F*^2^)] = 0.036	*w* = 1/[σ^2^(*F*_o_^2^) + (0.0459*P*)^2^ + 0.6636*P*] where *P* = (*F*_o_^2^ + 2*F*_c_^2^)/3
*wR*(*F*^2^) = 0.099	(Δ/σ)_max_ = 0.005
*S* = 1.08	Δρ_max_ = 0.61 e Å^−^^3^
1959 reflections	Δρ_min_ = −0.56 e Å^−^^3^
137 parameters	Extinction correction: SHELXL-2018/3 (Sheldrick 2015), Fc^*^=kFc[1+0.001xFc^2^λ^3^/sin(2θ)]^-1/4^
0 restraints	Extinction coefficient: 0.0045 (4)

### Special details

**Table:**

Geometry. All esds (except the esd in the dihedral angle between two l.s. planes) are estimated using the full covariance matrix. The cell esds are taken into account individually in the estimation of esds in distances, angles and torsion angles; correlations between esds in cell parameters are only used when they are defined by crystal symmetry. An approximate (isotropic) treatment of cell esds is used for estimating esds involving l.s. planes.

### Fractional atomic coordinates and isotropic or equivalent isotropic displacement parameters (Å^2^)

**Table:**

	*x*	*y*	*z*	*U*_iso_*/*U*_eq_	
Br	0.90737 (5)	0.85845 (3)	0.51410 (4)	0.0772 (2)	
O	0.6024 (3)	0.46212 (19)	0.28785 (15)	0.0704 (6)	
C1	0.5525 (4)	0.2291 (2)	0.3474 (2)	0.0524 (6)	
H1A	0.615715	0.217862	0.288281	0.063*	
H1B	0.424182	0.245293	0.311148	0.063*	
C2	0.5725 (5)	0.1238 (3)	0.4234 (3)	0.0646 (8)	
H2A	0.660804	0.070068	0.406688	0.077*	
H2B	0.455714	0.084094	0.411100	0.077*	
C3	0.6385 (5)	0.1665 (2)	0.5467 (2)	0.0573 (7)	
H3A	0.541287	0.160567	0.584874	0.069*	
H3B	0.742854	0.121317	0.589288	0.069*	
C3A	0.6916 (4)	0.2907 (2)	0.53807 (19)	0.0428 (5)	
N4	0.7726 (4)	0.35675 (17)	0.62262 (17)	0.0503 (5)	
C4A	0.8027 (3)	0.4710 (2)	0.59434 (19)	0.0424 (5)	
C5	0.8937 (4)	0.5471 (2)	0.6820 (2)	0.0569 (7)	
H5	0.932797	0.519968	0.757050	0.068*	
C6	0.9258 (4)	0.6610 (2)	0.6588 (2)	0.0554 (7)	
H6	0.986151	0.710837	0.717397	0.066*	
C7	0.8667 (4)	0.7005 (2)	0.5464 (2)	0.0490 (6)	
C8	0.7793 (4)	0.6286 (2)	0.4582 (2)	0.0486 (6)	
H8	0.742035	0.656476	0.383367	0.058*	
C8A	0.7471 (3)	0.5135 (2)	0.48227 (19)	0.0403 (5)	
C9	0.6560 (3)	0.4355 (2)	0.38867 (19)	0.0451 (5)	
N10	0.6368 (3)	0.32406 (18)	0.42522 (15)	0.0405 (4)	

### Atomic displacement parameters (Å^2^)

**Table:**

	*U*^11^	*U*^22^	*U*^33^	*U*^12^	*U*^13^	*U*^23^
Br	0.0940 (3)	0.0341 (2)	0.1020 (4)	−0.00671 (14)	0.0235 (2)	0.00799 (14)
O	0.1104 (17)	0.0553 (12)	0.0345 (9)	0.0020 (11)	0.0007 (10)	0.0064 (8)
C1	0.0651 (16)	0.0489 (15)	0.0402 (12)	−0.0045 (12)	0.0089 (11)	−0.0130 (11)
C2	0.090 (2)	0.0478 (16)	0.0519 (16)	−0.0179 (15)	0.0121 (15)	−0.0119 (12)
C3	0.088 (2)	0.0401 (13)	0.0432 (13)	−0.0170 (13)	0.0171 (13)	−0.0026 (11)
C3A	0.0572 (14)	0.0365 (12)	0.0347 (11)	−0.0038 (10)	0.0121 (10)	0.0004 (9)
N4	0.0768 (15)	0.0375 (12)	0.0338 (10)	−0.0096 (9)	0.0099 (10)	−0.0008 (8)
C4A	0.0548 (13)	0.0338 (12)	0.0377 (11)	−0.0018 (10)	0.0110 (10)	0.0000 (9)
C5	0.0807 (19)	0.0420 (14)	0.0412 (12)	−0.0082 (13)	0.0047 (12)	−0.0026 (11)
C6	0.0657 (16)	0.0411 (13)	0.0557 (15)	−0.0073 (12)	0.0100 (13)	−0.0097 (11)
C7	0.0530 (14)	0.0315 (12)	0.0638 (15)	0.0004 (10)	0.0177 (12)	0.0019 (11)
C8	0.0595 (15)	0.0385 (13)	0.0476 (13)	0.0059 (10)	0.0142 (11)	0.0086 (10)
C8A	0.0473 (12)	0.0363 (12)	0.0375 (11)	0.0042 (9)	0.0116 (9)	0.0011 (9)
C9	0.0571 (14)	0.0407 (13)	0.0354 (11)	0.0060 (10)	0.0090 (10)	0.0027 (9)
N10	0.0515 (11)	0.0373 (10)	0.0317 (9)	−0.0017 (8)	0.0093 (8)	−0.0033 (8)

### Geometric parameters (Å, º)

**Table:**

Br—C7	1.900 (3)	C3A—N10	1.371 (3)
O—C9	1.217 (3)	N4—C4A	1.392 (3)
C1—N10	1.471 (3)	C4A—C8A	1.396 (3)
C1—C2	1.503 (4)	C4A—C5	1.404 (3)
C1—H1A	0.9700	C5—C6	1.375 (4)
C1—H1B	0.9700	C5—H5	0.9300
C2—C3	1.522 (4)	C6—C7	1.389 (4)
C2—H2A	0.9700	C6—H6	0.9300
C2—H2B	0.9700	C7—C8	1.371 (4)
C3—C3A	1.494 (4)	C8—C8A	1.391 (3)
C3—H3A	0.9700	C8—H8	0.9300
C3—H3B	0.9700	C8A—C9	1.464 (3)
C3A—N4	1.290 (3)	C9—N10	1.376 (3)
			
N10—C1—C2	104.5 (2)	N4—C4A—C5	118.8 (2)
N10—C1—H1A	110.8	C8A—C4A—C5	118.3 (2)
C2—C1—H1A	110.8	C6—C5—C4A	121.1 (2)
N10—C1—H1B	110.8	C6—C5—H5	119.4
C2—C1—H1B	110.8	C4A—C5—H5	119.4
H1A—C1—H1B	108.9	C5—C6—C7	118.9 (2)
C1—C2—C3	107.0 (2)	C5—C6—H6	120.6
C1—C2—H2A	110.3	C7—C6—H6	120.6
C3—C2—H2A	110.3	C8—C7—C6	121.8 (2)
C1—C2—H2B	110.3	C8—C7—Br	119.1 (2)
C3—C2—H2B	110.3	C6—C7—Br	119.1 (2)
H2A—C2—H2B	108.6	C7—C8—C8A	119.0 (2)
C3A—C3—C2	105.3 (2)	C7—C8—H8	120.5
C3A—C3—H3A	110.7	C8A—C8—H8	120.5
C2—C3—H3A	110.7	C8—C8A—C4A	120.8 (2)
C3A—C3—H3B	110.7	C8—C8A—C9	119.6 (2)
C2—C3—H3B	110.7	C4A—C8A—C9	119.6 (2)
H3A—C3—H3B	108.8	O—C9—N10	121.4 (2)
N4—C3A—N10	125.3 (2)	O—C9—C8A	125.6 (2)
N4—C3A—C3	125.9 (2)	N10—C9—C8A	112.97 (19)
N10—C3A—C3	108.8 (2)	C3A—N10—C9	123.4 (2)
C3A—N4—C4A	115.8 (2)	C3A—N10—C1	113.1 (2)
N4—C4A—C8A	122.9 (2)	C9—N10—C1	123.41 (19)
			
N10—C1—C2—C3	9.8 (4)	C5—C4A—C8A—C8	−0.7 (4)
C1—C2—C3—C3A	−11.4 (4)	N4—C4A—C8A—C9	−1.2 (4)
C2—C3—C3A—N4	−172.4 (3)	C5—C4A—C8A—C9	178.5 (2)
C2—C3—C3A—N10	8.8 (3)	C8—C8A—C9—O	−0.4 (4)
N10—C3A—N4—C4A	0.9 (4)	C4A—C8A—C9—O	−179.6 (3)
C3—C3A—N4—C4A	−177.8 (3)	C8—C8A—C9—N10	178.8 (2)
C3A—N4—C4A—C8A	1.0 (4)	C4A—C8A—C9—N10	−0.3 (3)
C3A—N4—C4A—C5	−178.7 (3)	N4—C3A—N10—C9	−2.6 (4)
N4—C4A—C5—C6	−179.6 (3)	C3—C3A—N10—C9	176.2 (2)
C8A—C4A—C5—C6	0.7 (4)	N4—C3A—N10—C1	178.4 (3)
C4A—C5—C6—C7	0.0 (5)	C3—C3A—N10—C1	−2.7 (3)
C5—C6—C7—C8	−0.7 (4)	O—C9—N10—C3A	−178.6 (3)
C5—C6—C7—Br	179.0 (2)	C8A—C9—N10—C3A	2.1 (3)
C6—C7—C8—C8A	0.8 (4)	O—C9—N10—C1	0.3 (4)
Br—C7—C8—C8A	−179.02 (19)	C8A—C9—N10—C1	−179.0 (2)
C7—C8—C8A—C4A	−0.1 (4)	C2—C1—N10—C3A	−4.5 (3)
C7—C8—C8A—C9	−179.2 (2)	C2—C1—N10—C9	176.5 (2)
N4—C4A—C8A—C8	179.6 (2)		
